# Structural Basis for Resistance to Diverse Classes of NAMPT Inhibitors

**DOI:** 10.1371/journal.pone.0109366

**Published:** 2014-10-06

**Authors:** Weiru Wang, Kristi Elkins, Angela Oh, Yen-Ching Ho, Jiansheng Wu, Hong Li, Yang Xiao, Mandy Kwong, Mary Coons, Bobby Brillantes, Eric Cheng, Lisa Crocker, Peter S. Dragovich, Deepak Sampath, Xiaozhang Zheng, Kenneth W. Bair, Thomas O'Brien, Lisa D. Belmont

**Affiliations:** 1 Genentech, Inc., South San Francisco, California, United States of America; 2 Forma Therapeutics, Inc., Watertown, Massachusetts, United States of America; University of Washington, United States of America

## Abstract

Inhibiting NAD biosynthesis by blocking the function of nicotinamide phosphoribosyl transferase (NAMPT) is an attractive therapeutic strategy for targeting tumor metabolism. However, the development of drug resistance commonly limits the efficacy of cancer therapeutics. This study identifies mutations in NAMPT that confer resistance to a novel NAMPT inhibitor, GNE-618, in cell culture and *in vivo*, thus demonstrating that the cytotoxicity of GNE-618 is on target. We determine the crystal structures of six NAMPT mutants in the apo form and in complex with various inhibitors and use cellular, biochemical and structural data to elucidate two resistance mechanisms. One is the surprising finding of allosteric modulation by mutation of residue Ser165, resulting in unwinding of an α-helix that binds the NAMPT substrate 5-phosphoribosyl-1-pyrophosphate (PRPP). The other mechanism is orthosteric blocking of inhibitor binding by mutations of Gly217. Furthermore, by evaluating a panel of diverse small molecule inhibitors, we unravel inhibitor structure activity relationships on the mutant enzymes. These results provide valuable insights into the design of next generation NAMPT inhibitors that offer improved therapeutic potential by evading certain mechanisms of resistance.

## Introduction

There is considerable interest in using inhibitors of nicotinamide phosphoribosyl transferase (NAMPT, PBEF, or visfatin) for cancer therapy [1]. Three NAMPT inhibitors of two distinct structural classes have entered clinical trials for oncology indications, APO866 (formerly FK866) [2], [3], GMX1778 (formerly CHS-828) and a pro-drug of GMX1778, GMX1777 [4]–[8]. NAMPT functions as a homo-dimeric enzyme that catalyzes the rate limiting step in the primary salvage pathway for NAD synthesis, the transfer of a phosphoribosyl residue from 5-phosphoribosyl-1-pyrophosphate (PRPP) to nicotinamide (NAM) to produce nicotinamide mononucleotide (NMN) [9]–[11]. Inhibition of NAMPT causes rapid cellular depletion of NAD [2], [7], [12], [13], an essential cofactor for ATP production as well as a substrate for poly(ADP-ribose) polymerases and sirtuins. Cancer cells are expected to be more sensitive to NAMPT inhibition than normal tissue due to their high metabolic requirement and increased dependence upon NAD consuming enzymes [14]. Furthermore, it has been proposed that the therapeutic index for NAMPT inhibitors can be increased in patients whose cancers lack NAPRT1, an essential enzyme in an alternative NAD synthesis pathway that utilizes nicotinic acid (NA, naicin, vitamin B3) as a starting point [15]. Supplementation with NA can relieve NAMPT inhibitor toxicity in animal models, allowing higher doses of NAMPT inhibitors to be tolerated [12], [16].

**Figure 1:**
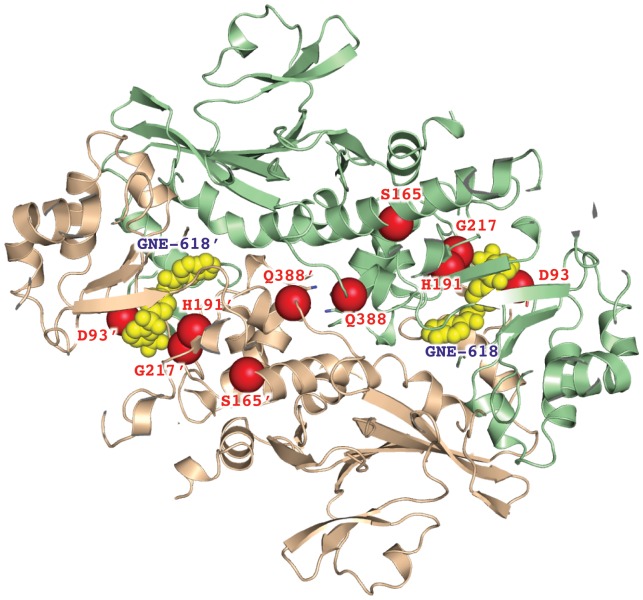
Crystal structure of NAMPT with resistant mutations mapped. The NAMPT protein structure is shown in ribbon diagram displaying an active dimer. The monomers of the NAMPT dimer are colored in brown and green, respectively. Two inhibitor molecules of GNE-618 are bound to this structure and are shown as yellow spheres. Some of the resistant mutations are mapped on the structure and are shown as red spheres.

Crystal structures of NAMPT in numerous ligand-bound forms have been reported [17]–[21]. These structures consistently display a NAMPT homo-dimer with two essentially identical active sites at the dimer interface (Figure 1). Typical NAMPT inhibitors were found to occupy the portion of active site responsible for NAM binding and a tunnel-shaped cavity extending from the NAM binding site. A distinct feature of many NAMPT inhibitors is the dependence on nitrogen-containing heterocyclic moieties to achieve cellular potency [12], [18]–[21]. As the inhibitors bind to the NAMPT protein, those heterocyclic moieties protrude into the NAM binding site and mimic the natural substrate to form covalent adducts with PRPP.

NAMPT mutations that confer resistance to GMX1778, APO866 or TP201565, a structural analog of GMX1778 have been mapped to G217R, H191R, D93del, and Q388R [12], [22]. Based on the wild-type enzyme structure, residues G217R and H191R appeared to protrude into the inhibitor-binding pocket, while D93 and Q388 are located on the dimer interface. In this work, we identify and characterize six mutations in NAMPT that confer resistance to a novel small molecule inhibitor of NAMPT, GNE-618. These include G217R, D93del as well as 4 previously unreported mutations. Furthermore, we determine the crystal structures of six NAMPT mutants in the apo form and in complex with various inhibitors and present a definitive model to explain the differential effects of the mutations on various structural classes of NAMPT inhibitors.

## Results

### Selection and characterization of GNE-618 resistant cell lines

GNE-618 is a novel small molecule that potently inhibits NAMPT activity and exhibits efficacy in xenograft models of cancer [21], [23]. A proposed clinical strategy for NAMPT inhibitor development includes selection of patients with NAPRT1-deficient tumors and co-administration of NA. Thus, a potential mechanism of resistance to NAMPT inhibitors in NAPRT1-deficient cancer is NAPRT1 re-expression in the presence of co-administered NA. To test this hypothesis, we modeled resistance in cell lines that lack NAPRT1 gene expression and selected for resistance in the absence or presence of 10 µM NA. By culturing cell lines in increasing concentrations of GNE-618, we obtained cells that proliferated in GNE-618 concentrations 100 fold higher than the IC_50_ of the corresponding NAPRT1 deficient parental cell lines (RD, MiaPaCa-2, NCI-H460) or the NAPRT1 proficient NCI-H520 cell line. Short tandem repeat (STR) profiling of the resistant cell lines matched the parental cell lines (data not shown), indicating that they were derived from that cell line.

NAPRT1 deficient cell lines did not re-express NAPRT1 even when grown in the presence of 10 uM NA, suggesting that this mechanism of resistance is not common in cultured cells. We observe an increase in NAMPT levels in some cell lines, most notably in the RD cell line, consistent with reports that higher levels of NAMPT correlate with decreased sensitivity to NAMPT inhibitors [12], [23]. However, increased NAMPT was not observed in all cell lines, suggesting alternative mechanisms of resistance (Figure S1a).

### GNE-618 resistant cell lines harbor a variety of NAMPT mutations

**Figure 2:**
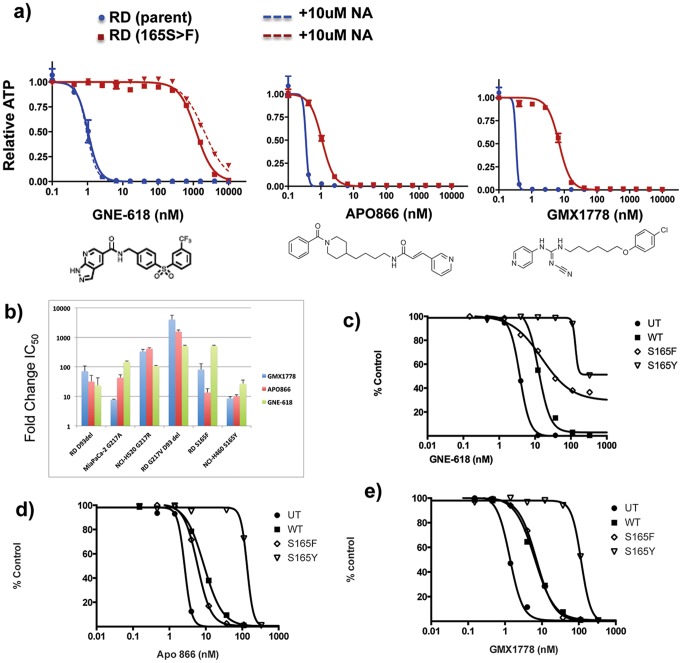
Characterization of GNE-618 resistant cell lines. a) Example IC_50_ of RD parent versus the resistant derivative line harboring the S165F NAMPT mutation in the absence (solid lines) or presence (dashed line) of 10 µM NA. b) Fold shifts in absolute IC_50_ values in resistant versus parental cell lines. Error bars represent the standard deviation of three independent runs. c-e) NAMPT S165F and S165Y were expressed in 293T cells and evaluated for response to c) GNE-618, d) APO866 and e) GMX1778. WT = wild-type NAMPT, UT = untransfected.

DNA sequencing of *NAMPT* from resistant cell lines identified G217R and D93 deletion as well as four novel mutations, G217A, G217V, S165F, and S165Y (Table 1). While G217 is close to the inhibitor-binding pocket (Figure 1), S165 is further away from the binding pocket and was not previously identified as a resistance mutation in studies of GMX1778 or APO866. The RD cell line with the S165F mutation is almost 1000-fold resistant to GNE-618, but only10-fold and 100-fold resistant to APO866 and GMX1778, respectively, demonstrating that inhibitors from distinct classes are differentially affected. We also note that the IC_50_ for GNE-618 was not significantly different in the presence or absence of 10 µM NA (Figure 2a). The same observation was made for all of the NAPRT1 deficient cell lines, consistent with our conclusion that the NAPRT1 pathway was not re-activated as a resistance mechanism.

**Table 1:** Nampt mutations Identified in Resistant Cell Lines.

Cell Line	Tissue Diagnosis	NA	Parental GNE-618 IC_50_ (nM)	NAMPT Mutation	NAPRT1 Status
RD	rhabdomyosarcoma	-	2±0.2	D93del, G217V	Negative
RD	rhabdomyosarcoma	+	2±0.2	S165F	Negative
NCI-H460	large cell lung cancer	+	197±6	S165Y	Negative
Mia-Paca2	Pancreatic carcinoma	+	34±2	G217A	Negative
NCI-H520	Squamous lung carcinoma	-	9±0.4	G217R	Positive

Cell lines that were selected to grow in the presence of 100 fold the parental IC_50_ for GNE-618. NA indicates whether the cell line was selected in the presence or absence of 10 uM nicotinic acid. NAPRT1 status did not change as a result of selection for GNE-618 resistance.

Cell lines harboring S165F, S165Y, or G217A are preferentially resistant to GNE-618 compared to GMX1778 and APO866. In contrast, NCI-H520 cells harboring the G217R mutant are similarly resistant to all 3 NAMPT inhibitors (Figure 2b). The RD line harboring the G217V mutation also had the D93 deletion. An earlier passage of the cell line harbors only the D93 deletion, and is 25 to 70 fold resistant to the NAMPT inhibitors. The D93del/G217V RD line is 500 to 4000 fold resistant suggesting that a significant increase in resistance is conferred by the G217V mutation. A minimum of 90% reduction in cellular NAD levels is required for cell killing by NAMPT inhibition [23], [24]. We find that all of the resistant lines with the exception of the lines harboring only D93del or S165Y result in less than 90% reduction of NAD after exposure to GNE-618 at 100-fold the parental line IC_50_ (Figure S1 b–c). These data are consistent with the observation that the IC_50_ values of GNE-618 for cell lines carrying either D93del or S165Y are shifted less than 100 fold, while cell lines harboring the other mutations exhibit at least a 100 fold increase in GNE-618 IC_50_.

The identification of resistance mutations in S165 was surprising because this residue does not lie within the drug-binding pocket. To confirm that the S165Y/F mutations were sufficient for resistance, we expressed the mutant and wild-type proteins in a naïve, sensitive cell line. As expected, wild-type NAMPT caused a reduction in sensitivity to NAMPT inhibitors, however, both S165F and S165Y mutants resulted in diminished sensitivity to GNE-618 compared to wild-type (Figure 2c). The effects of the S165F/Y mutant NAMPT proteins on sensitivity to GMX1778 and APO866 were more modest, consistent with the data obtained from the selected resistant cells (Figure 2d,e). In all cases the S165Y mutant resulted in greater resistance to NAMPT inhibitors.

### S165Y mutation confers resistant to NAMPT inhibition *in vivo*

**Figure 3:**
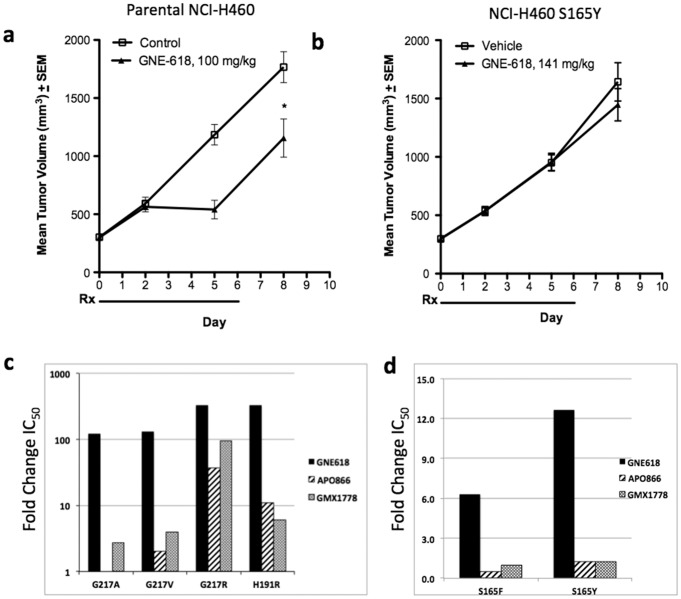
Efficacy of GNE-618 in the S165Y NCI-H460 xenograft model. a–b) Mean tumor volume + standard error of the mean (SEM) in NCI-H460 parental (a) and NCI-H460 S165Y mutant (b) xenograft models after treatment with GNE-618 at the doses indicated. c) Fold change in IC_50_ of NAMPT inhibitors on purified mutant NAMPT proteins bearing mutations in G217 and H191 compared to wild-type enzyme; d) fold change in IC_50_ of NAMPT inhibitors on purified mutant NAMPT proteins bearing mutations in S165 compared to wild-type enzyme. Rx  =  treatment period. *p = 0.007 compared to vehicle control.

Treatment of mice bearing NCI-H460 tumor xenografts with GNE-618 (100 mg/kg) for 7 continuous days (QD) by oral administration resulted in 54% tumor growth inhibition (Figure 3a; p = 0.007 vs. vehicle), consistent with previous studies [21], [23]. In contrast, NCI-H460 S165Y tumor xenografts were completely resistant to treatment with GNE-618, even when dosed daily (QD) at higher drug concentrations (Figure 3b). The doses of GNE-618 tested in both NCI-H460 parental and S165Y mutant models were well tolerated as no significant weight loss, morbidity or mortality was noted throughout the study.

### Resistance mutations differentially impact inhibitor compound classes

We next engineered the NAMPT mutations in *E. coli* expression constructs, purified the mutant proteins and evaluated response to GNE-618, APO866 and GMX1778. The H191R and all G217 mutant NAMPT proteins exhibited at least 100- fold increases in GNE-618 IC_50_ compared to wild-type. The effects on GMX1778 and APO866 were more varied, with G217R and H191R exhibiting the largest shifts and G217V and G217A showing more modest shifts in GMX1778 and APO866 IC_50_ values (Figure 3c, Table 2). The S165 mutants exhibited smaller shifts in IC_50_ and are therefore plotted on a different scale. The S165 mutants were less sensitive to GNE-618, but had similar sensitivity to GMX1778 and APO866 compared to wild-type (Figure 3d, Table 2).

**Table 2:** Biocheµical IC_50_Values of Structurally Diverse NAMPT inhibitors.

Cpmd	WT	WT (sd)	G217V	G217V (sd)	G217R	G217R (sd)	G217A	G217A (sd)	S165F	S165F (sd)	S165Y	S165Y (sd)	H191R	H191R (sd)
1	0.015	0.004	1.962	0.679	>5	NA	1.844	0.946	0.095	0.033	0.190	0.085	>5	
2	0.005	0.000	>5	NA	>5	NA	0.191	0.007	0.015	0.000	0.021	0.003	2.170	0.687
3	0.010	0.003	0.980	0.323	>5	NA	0.344	0.033	0.038	0.012	0.081	0.028	3.004	1.179
4	0.080	0.025	4.155	3.926	>5	NA	>5	NA	1.933	0.985	2.528	1.967	>5	NA
5	0.022	0.012	3.124	0.764	>5	NA	>5	NA	0.195	0.076	0.326	0.171	>5	NA
6	0.026	0.017	>5	NA	>5	NA	>5	NA	0.175	0.098	0.148	0.103	>5	NA
7	0.007	0.006	3.380	2.787	>5	NA	2.052	0.004	0.042	0.014	0.115	0.004	>5	NA
8	0.010	0.004	2.130	0.524	>5	NA	0.658	0.071	0.029	0.010	0.075	0.025	2.156	0.814
9	0.004	0.001	0.008	0.003	0.147	0.066	0.004	0.003	0.002	0.001	0.005	0.002	0.044	0.012
10	0.003	0.001	0.060	0.008	>5	NA	0.010	0.000	0.003	0.001	0.005	0.003	0.611	0.400
11	0.004	0.001	0.016	0.003	0.385	0.224	0.011	0.004	0.004	0.002	0.005	0.000	0.024	0.021
12	0.007	0.003	2.678	0.795	>5	NA	0.180	0.040	0.017	0.001	0.025	0.018	0.438	0.034

Values are in umol/L, sd =  standard deviation, NA = not applicable.

**Figure 4:**
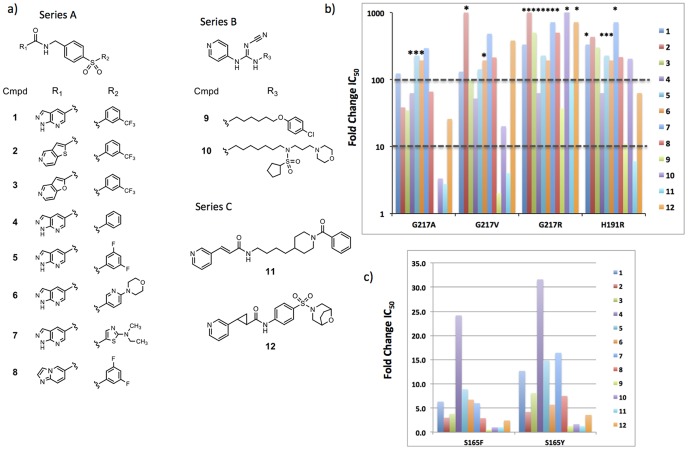
Nampt inhibitors evaluated for potency on mutant NAMPT enzymes. a) Chemical structures of NAMPT inhibitors, compound 1 is GNE-618, compound 9 is GMX1778, compound 11 is APO866. b) Fold change in IC_50_ of NAMPT inhibitors on purified mutant NAMPT proteins compared to wild-type enzyme. c) Fold change in IC_50_ values for S165Y/F mutants compared to wild-type. * fold-change assuming IC_50_  =  maximum concentration tested (5 µM). Actual fold-change is likely larger than that depicted.

To expand the structure activity relationship, we profiled the mutant enzymes against a larger, structurally diverse panel of NAMPT inhibitors (Figure 4). These included compounds with a diaryl-sulfone moiety at one molecular terminus and a bicyclic heterocycle at the other (Series A) [18], [19], [21]. Other structurally-distinct compounds which incorporated cyanoguanidine groups were also utilized in these assessments (Series B) [25] as well as a molecule bearing a *trans*-cyclopropane moiety [26] (compound 12). Overall, the pattern of sensitivity to the various NAMPT mutations appears to depend primarily on the structure of the compound core and not the appended R1 and R2 groups (Figure 4 b–c, Table 2). Compounds with a bulky phenyl group in the core (series A and compound 12) were less active on all G217 mutants, while compounds with a narrow more flexible linker (series B and compound 11) maintain much of their potency on the more conservative changes of G217A and G217V.

### S165F/Y mutations unwind a helix in NAMPT active site

**Figure 5:**
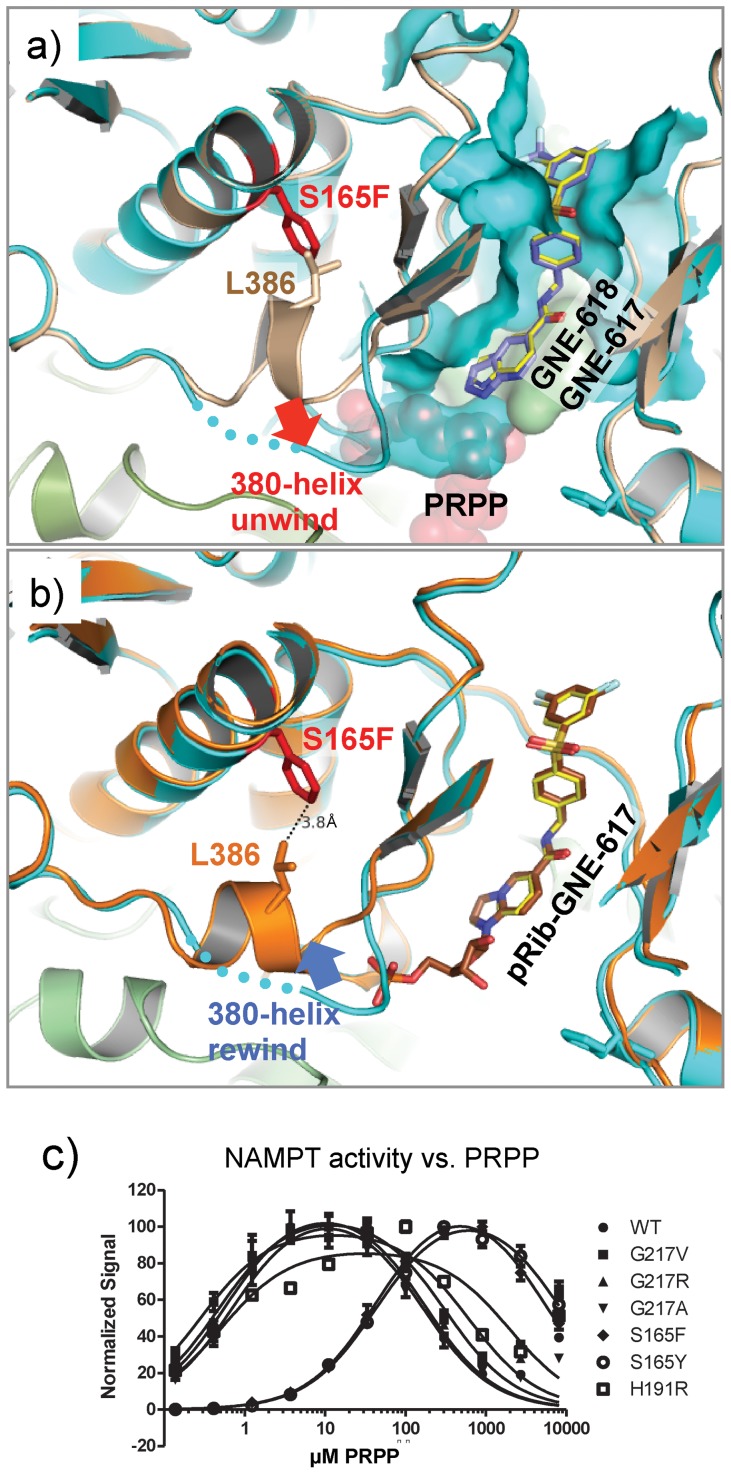
Conformational changes in 380-helix induced by resistant mutations at S165. a) Superimposition of NAMPT-S165F/GNE-618 complex structure (ribbons colored in cyan) onto a wild-type apo-structure (PDB entry 4KFO, ribbons colored in brown). GNE-618 is shown in sticks, and colored by atom type (carbons in light blue). The inhibitor-binding site is drawn in surface representation, two monomers colored in cyan and green, respectively. S165F is shown in red sticks, which imposes a steric clash onto residue L386 (sticks colored in wheat) of the wild-type structure. The normal PRPP binding mode is illustrated in transparent red spheres. For comparison, a structure of GNE-617 is shown in sticks (carbons in yellow). b) Superimposition of NAMPT-S165F/pRib-GNE-617 complex (ribbons colored in orange) onto the structure of NAMPT-S165F/GNE-617 ribbons colored in cyan). The pRib-GNE-617 molecule is shown in sticks (carbons in brown). GNE-617 is also shown in sticks (carbons in yellow). S165F is shown in red sticks. The resulting 380-helix avoided the steric clash between with Phe165. c) NMN production by wild-type (WT) and mutant NAMPT enzymes in the indicated concentrations of PRPP.

In order to understand the structure activity relationship between the various NAMPT inhibitors and these resistance mutants, we solved the crystal structures of six mutant NAMPT proteins. The crystal structures of NAMPT S165F/Y revealed a somewhat surprising conformational change in a small helical structural motif spans the sequence G_383_GGLLQ_388_ (Figure 5a). We designate this motif as “380GRS” to reflect its position in the primary sequence, and its glycine rich characteristic. As the structural consequences of S165F and S165Y are essentially identical, we focus on the S165F structure in the following discussion. A survey of previously determined crystal structures of NAMPT indicated that 380GRS consistently adopted an α-helical structure regardless of substrate binding status (apo - PDB entry 2H3B [27], NMN bound - PDB entry 2GVG [28], PRPP bound - PDB entry 2E5C [29]). A helical structure of 380GRS is crucial for PRPP binding, as it is situated in the PRPP binding site and the electric dipole moment developed in the helix can complement the negative charges associated with the PRPP 5′-phosphate group. This is one of the common phosphate-binding motifs observed in various protein structures [30]. The unwinding of the 380GRS in S165 mutants rendered the structure less compatible with the binding of PRPP (Figure 5a). However, expression of S165F/Y mutant NAMPT allows cell survival in the presence of GNE-618, implying that the mutant NAMPT retains a level of enzymatic activity to support sufficient NAD production for cell survival. Our structural studies thus reveal that the S165 mutation modulates NAMPT activity by interfering with PRPP binding in an allosteric manner.

The fact that S165 mutants are resistant to the inhibitors suggested 380GRS is critical to inhibitory mechanism. However, the part of NAMPT structure that is responsible for inhibitor binding is not affected by the S165 mutations. This prompted us to investigate whether the mutations had effects on the phosphoribosylated (pRib) adduct of the inhibitors, because it has been shown that the cellular potency of NAMPT inhibitors is dependent on the formation of a pRib adduct and the resulting inhibitory activity of the compound adduct [31]. We determined the crystal structures of NAMPT-S165F in complex with GNE-617 (Figure S2), a close analogue of GNE 618, and its adduct pRib-GNE-617. These structures revealed that the pRib-GNE-617 binding converted 380GRS back into a helical conformation (Figure 5b). The rewound helix, however, adopted a different orientation compared to the wild-type structure and avoided the steric clashed between Leu386 and Phe165. The dramatic conformational changes induced by binding the pRib-adduct suggested a significant energetic barrier and decreased inhibitory activity. Because the S165S/F mutants impacted the PRPP binding site, we evaluated the requirement for PRPP for enzymatic activity in the mutant proteins. Consistent with the structural observations, S165F/Y mutant NAMPT proteins required approximately 500 fold more PRPP compared to the wild-type protein and the other NAMPT mutants (Figure 5c).

### H191 and G217 mutations interfere with inhibitor binding by steric clash

**Figure 6:**
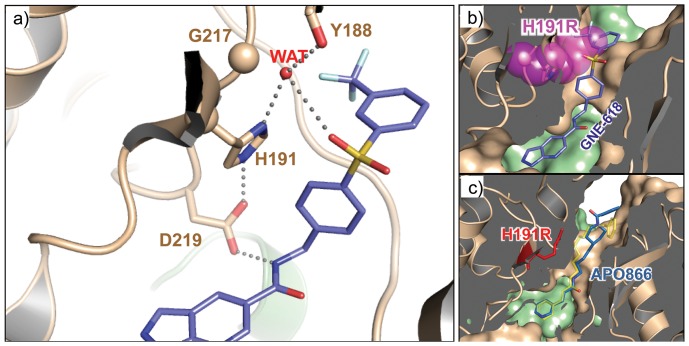
H191 derived resistance. a) A close-up view of NAMPT inhibitor binding site. GNE-618 is shown in sticks (carbon in blue). NAMPT is shown in ribbons diagram, and colored by monomers, brown and green, respectively. The key residues (Asp219, His191, Gly217, Tyr188) forming hydrogen bond network are shown in sticks (carbon in brown). A water molecule WAT mediating hydrogen bonds is shown as a red sphere, dotted lines are hydrogen bonds. **b**) The structure of NAMPT in complex with GNE-618. GNE-618 is shown in sticks and colored by atom-type (carbons in blue). NAMPT is shown in both ribbons and surface rendering, with monomers in brown and green, respectively. The interior of the NAMPT protein is blinded in gray. H191 side chain is shown in sticks. Mutation H191R side chain is plotted in sticks and transparent spheres, in magenta. **c**) Complex structure of APO866 with NAMPT-H191R. APO866 is shown in sticks (blue for carbons). For comparison, APO866 conformation in wild-type NAMPT is shown in transparent sticks, colored in yellow. The protein is shown in both surface rendering and ribbons. The monomers are in brown and green, respectively. The interior of the NAMPT protein is blinded in gray. The His191 side chain is shown in red sticks.

We next investigated the structural consequences of mutations at H191 and G217. As depicted in Figure 6a, H191 and G217 together with D219 and Y188 form one side of the tunnel wall surrounding the cavity commonly occupied by NAMPT inhibitors. This proximity suggests that these mutations could directly interfere with GNE-618 binding. The H191 imidazole ring forms a herringbone stacking interaction with the GNE-618 phenyl linker moiety. The same interaction is observed for other bi-aryl sulfone inhibitors [18]-[20]. The stacking interaction defined the orientation of the inhibitor linker moiety inside the tunnel passage and oriented the inhibitor's terminal group to exit the tunnel. The optimal orientation of H191 imidazole ring is facilitated by hydrogen bond interactions with D219 and a water molecule (WAT), which in turn interacts with the main chain carbonyl of Y188 (Figure 6a). A previously published *in silico* model predicted that H191R would protrude its side chain into the tunnel and sterically block inhibitors like APO866 from binding [22]. When tested across a panel of structurally diverse inhibitors, H191R reduced potency across the compound families, but remained more sensitive to APO866 and GMX1778 than series A inhibitors (Figure 4, Table 2). To reconcile the discrepancy, we determined the crystal structure of NAMPT-H191R. The R191 side chain indeed occupied part of the volume inside the tunnel region, but did not completely block the tunnel passage (Figure 6b), thus imposing a more stringent limit on the size of linker moieties in the inhibitor molecules. The bi-aryl sulfone group of series A compounds exceeded the available space, whereas the more flexible and narrower linker of APO866 can fit through the altered tunnel (Figure 6c).

**Figure 7:**
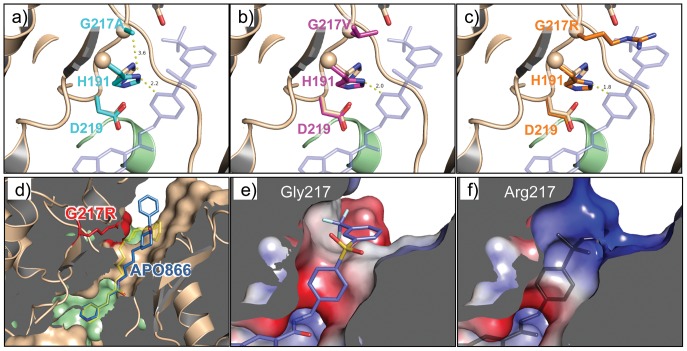
G217 derived resistance. a) Structure of NAMPT-G217A superimposed onto the wild-type structure in b) (the brown and green ribbons). Only the side chains of residues Asp219, His191, and Ala217 of the G217A mutant are shown (cyan) together with those of wild-type (brown) for comparison. The transparent blue sticks indicate the position of GNE-618 in wild-type structure. Note that Ala217 induced a rotation of the His191 imidazole ring. Similar effects were caused by the G217V mutant (magenta) and G217R mutant (orange) depicted in **b**) and **c**). d) The crystal structure of APO866 in complex with G217-NAMPT with similar representations as above. G217R side chain is shown in sticks, and colored in red. **e**) Complex structure of NAMPT with GNE-618 shown in surface rendering colored by electrostatic potential (blue positive, red negative, white neutral). GNE-618 is shown as sticks (carbon in blue). **f**) Structure of NAMPT G217R shown in surface rendering colored by electrostatic potential as in g). The transparent black sticks indicate the GNE-618 binding position in wild-type NAMPT.

The crystal structures of G217 mutants revealed that they also perturbed inhibitor binding through a His191-mediated mechanism. Glycine is required for this position to allow a water molecule to bind and participate in the D219-H191-WAT-Y188 network. The addition of a Cβ atom in G217A occludes the water molecule and disrupts the hydrogen bond network (Figure 7a). In addition, A217 pushes the H191 imidazole ring away from its normal orientation by 30°, rendering this part of the tunnel less compatible with GNE-618 binding. The conformational changes induced by G217A are amplified in the G217V mutant (Figure 7b), where the H191 side chain is twisted 40° away from the optimal herringbone conformation. Similar to H191R, inhibitors with narrow and flexible linkers, including APO866, are less sensitive to the relatively subtle changes induced by the G217A and G217V mutations, which is also consistent with the modest drop in the biochemical potency of compounds 9-11 (Figure 4, Table 2). Unlike the G217A/V mutations, the G217R mutation caused more drastic reduction in the inhibitory potency for all compounds on our test panel. G217R causes His191 side chain to rotate to the same extent as G217V, while introducing additional changes to the structure of the NAMPT inhibitor-binding site (Figure 7c). The R217 side chain competes for space with the bound inhibitor (e.g., with the terminal phenyl-trifluoromethyl group of GNE-618) in a similar fashion as H191R. The crystal structure of G217R in complex with APO866 revealed that the more flexible and narrower linker of APO866 adopted an alternative conformation but nonetheless could fit through the altered tunnel (Figures 7d). Besides introducing steric clashes, the R217 guanidinium group also creates a basic patch at the surrounding protein surface (Figures 7e–f) that favors polar groups over hydrophobic groups. We conclude that these additional structural changes render the G217R mutation more deleterious for NAMPT inhibitor binding across structural classes.

## Discussion

We identified and characterized a variety of NAMPT protein mutations mediating resistance against the bi-aryl sulfone inhibitors, exemplified by GNE-618. The identification of resistance mutations in S165 is unexpected given its distance from the inhibitor-binding site. However, expression of S165F or S165Y mutant NAMPT proteins in a naïve cell line resulted in decreased sensitivity to GNE-618, indicating that these mutations are sufficient to cause resistance to this NAMPT inhibitor. Furthermore, xenografts derived from cells harboring the S165Y mutation in NAMPT are resistant to GNE-618 at doses that are efficacious in the parental line, suggesting that this is relevant *in vivo*. Structural analyses of S165 NAMPT mutant proteins establish the critical role of the 380GRS in NAMPT catalysis. Crystal structures revealed a previously underappreciated conformational flexibility in this secondary structure element that can be exploited by resistance mutations through an allosteric mechanism. Our finding regarding the S165F/Y mechanism of action provides an explanation regarding another mutant, Q388R, previously reported to cause resistance to GMX1778. Q388 is located at the C-terminal end of the 380GRS, and is normally buried under the protein surface. It is unlikely for an arginine residue to settle in the same space and maintain the wild-type conformation of the helix. Given the flexibility associated with the 380GRS revealed by our analysis, we propose that R388 is likely to drive it into an alternative conformation that can negatively impact NAMPT inhibitor potency.

In conclusion, we present a systematic approach of identifying resistant mutations and elucidating their mechanism of action. Our work provides the most comprehensive to-date structural analysis of resistance mutations to NAMPT inhibitors, important for future rational drug design of more effective inhibitors against this target and similar classes of enzymes and for better understanding of the catalytic mechanism for this important metabolic enzyme.

## Materials and Methods

### Selection and Characterization of GNE-618 Resistant Cell Lines

Cell lines were obtained from the American Type Culture Collection (ATCC) or Deutsche Sammlung von Mikroorganismen und Zellkulturen (DSMZ) and stored in a central cell bank. Lines were authenticated by short tandem repeat (STR) and genotyped upon re-expansions and passaged no more than 20 times after thawing. IC_50_ measurements and western blots were performed as previously described [32].

### Animal models

All *in vivo* efficacy studies were approved by Genentech's Institutional Animal Care and Use Committee (IACUC) and adhered to the ILAR *Guide for the Care and Use of Laboratory Animals*. Nine to fourteen week-old female C.B-17 SCID beige mice (Charles River Laboratories) were inoculated subcutaneously with either 5 million NCI-H460 or 5 million NCI-H460 S156Y cells suspended in a 1∶1 ratio of HBSS and phenol red-free matrigel (BD Biosciences). Mice with similarly sized tumors (mean volume 200–300 mm^3^) were randomized into treatment cohorts. Mice were dosed for daily (QD) 7 days, orally, with vehicle [PEG400/H2O/EtOH (60/30/10; vol/vol/vol)] or GNE-618. Tumor sizes and body weights were recorded twice weekly. Tumors were measured using digital calipers and volumes were calculated using the formula *lw* ^2^×0.5 and reported as mean tumor volume ± SEM. Mice with tumor volumes ≥2000 mm^3^ or with losses in body weight ≥20% from their weight at the start of treatment were euthanized per IACUC guidelines. Percent tumor growth inhibition (TGI) was calculated using the formula [(mean tumor volume_vehicle_ − mean tumor volume_treatment_) ÷ mean tumor volume_vehicle_] ×100. Student's t-test was used to calculate significance in anti-tumor response between drug and vehicle treated mice.

### Generation and Purification of Mutant NAMPT Proteins for Biochemical Characterization

An expression construct for full length human (XP_540386) NAMPT was constructed using pET21B, a gift from professor Yigong Shi's group (Tsinghua University, Beijing, China). Mutations at positions S165F, S165Y, H191R, G217A, G217R, and G217V were introduced by site-directed mutagenesis. Wild-type and mutant proteins were expressed as C-terminal His6 fusion proteins in *E. coli*. Expression was induced in Rosetta (DE3) cells at 260 rpm and 22°C overnight using 1.0 mM IPTG. Cells were harvested by centrifugation at 4000g for 15 min. at 4°C.

Cell pellets were suspended in Lysis buffer A (50mM Tris/HCl, pH8.0, 300mM NaCl, 20mM Imidazole, 1mM TCEP (Tris(2-carboxyethyl)phosphine hydrochloride) and 1X protease inhibitor cocktail Complete (Roche). The Cell suspension was disrupted by passing through a microfluidizer (Model 110F Microfuidizer Cooperator) three times. The whole cell lysate was centrifuged at 40,000 rpm (Beckman Ti45 rotor) for 1 hour at 4°C. The supernatant was applied to a Ni-NTA SuperFlow column (Qiagen), washed extensively with wash buffer B (20mM Tris/HCl, pH8.0, 300mM NaCl, 20mM Imidazole, 0.5mM TCEP). NAMPT was eluted with elution buffer C (20mM Tris/HCl, pH8.0, 300mM NaCl, 250mM Imidazole, 0.5mM TCEP). Fractions containing NAMPT were further purified with Superdex 200 column (Amersham) pre-equilibrated with buffer D (20mM Tris/HCl, ph8.0, 150mM NaCl, 10% Glycerol, 0.5mM TCEP). The purified NAMPT was greater than 95% pure assessed by SDS-PAGE. The concentration of the purified protein was determined with Bradford protein assay.

### Biochemical Characterization of Mutant NAMPT Proteins

NAMPT enzymatic reactions were carried out in Buffer A (50 mM Hepes pH 7.5, 50 mM NaCl, 5 mM MgCl2, and 1 mM Tris(hydroxypropyl)phosphine (THP)). For compound inhibition studies the final concentration of substrates was 1 µM NAM, 100 µM PRPP, and 2.5 mM ATP. To obtain sufficient NMN levels for detection, we used WT, G217V, G217R, G217A, S165F, and H191R protein at a final concentration of 30 nM, and S165Y protein at 50 nM. 89 µL of enzyme was added to 1 µL 100X compound in DMSO, incubated for 15 minutes at room temperature, the reaction was started with the addition of 10 µL10x substrate solution. After 40 minutes, the reaction was quenched with 10 µL of 10% formic acid and 100 µM L-Cystathionine. Normalized data were analyzed using GraphPad Prism (version 5.04 for Windows, GraphPad Software, www.graphpad.com). A mass spectrometry-based assay was used to measure the NAMPT reaction product (NMN) and the internal control (L-Cystathionine) using an Agilent RapidFire/MS system. Analytes were bound to a graphitic carbon cartridge in 0.1% formic acid, eluted in 30% acetonitrile buffer, and injected into a Sciex 4000 mass spectrometer. The analytes in the sample were ionized with electrospray ionization and the positive ions were detected. The Q1 (parent ion) and Q3 (fragment ion) masses of NMN were 334.2 and 123.2, respectively. The Q1 and Q3 for L-Cystathionine were 223.1 and 134.1, respectively. Phospho-Ribose adduct formation assays and NAMPT enzymatic reactions were performed as previously described[19].

### Purification of mutant NAMPT protein for crystalization

C-terminal His-tagged NAMPT M1-H149 wild-type and mutant proteins with various single mutations were purified and crystallized by similar methods. M1-H491 constructs were transformed into BL21 codon optimized cells. Cells were grown at 37°C to an absorbance of 0.5 (OD_600_) in LB media containing 50 µg/mL of carbenicillin and then transferred to 22°C prior to induction with 1mM IPTG at an absorbance of 0.8 (OD_600_). Cells were harvested 16 hours post induction and the pellet was lysed by passing through a microfluidizer in a buffer containing 20 mM TrisCl, pH 8.0, 200 mM NaCl, 5 mM Imidazole, 1 mM TCEP and EDTA-free protease inhibitor tablets (Roche). Cell lysates were loaded onto a HisTrap HP column (GE Healthcare) in a buffer containing 20 mM TrisCl, pH 8.0, 500 mM NaCl, 5 mM Imidazole and 1 mM TCEP and the bound NAMPT was eluted with 250 mM Imidazole in the same buffer. The protein was then loaded onto a 5 mL Q HP column (GE Healthcare) in a buffer containing 25 mM TrisCl, pH 8.0 and 1 mM TCEP and eluted at 300 mM NaCl with a linear gradient of the same buffer and 1.0 M NaCl. The protein was further purified on a size-exclusion Superdex 75 column (GE Healthcare) in a buffer containing 25 mM TrisCl, pH 8.0, 150 mM NaCl and 5 mM DTT. Fractions containing NAMPT proteins were pooled and concentrated to 6 mg/mL and confirmed by SDS-PAGE showing that the purity was greater than 95%.

### Crystallization of NAMPT mutants

Diffraction quality crystals for all the proteins were grown at 19°C from 0.2 uL+0.2 uL vapor diffusion drops containing 6mg/mL NAMPT, 0.1 M Sodium phosphate, pH 8.6–9.0, 25–29% polyethylene glycol 3350 and 0.2 M NaCl, which were then streak-seeded using Hampton's seeding tool. Plate crystals appeared overnight and harvested for data collection with 15% ethylene glycol in the crystallization solution. For the co-structure of NAMPT with phosphoribosyl adduct of GNE-617, apo crystals were transferred into a buffer containing 0.1 M TrisHCl, 25% polyethylene glycol 3350, 0.2 M NaCl, 5 mM MgCl_2_ and 100 uM 5-phospho-D-ribose 1-disphosphate pentasodium salt (PRPP) and soaked over night at 19°C. The soaked crystals were then harvested with 15% ethylene glycol in the soaking solution without the PRPP and MgCl.

### X-ray data collection and structure determination

The diffraction data sets were collected at various beam lines and processed with the HKL2000 package [33] and XDS [34]. Further processing was carried out with programs from the CCP4 suite. Data collection and structure refinement statistics are summarized in Table 3. The structure was solved by molecular replacement (MR) with known Nampt structure (PDB code:3DHF [35]) as the search model using the program Phaser. Manual model building was done using program COOT[36]. The structure was further refined using program REFMAC5 [37] and PHENIX [38] using the maximum likelihood target functions, anisotropic individual B-factor refinement method, and TLS refinement method, to achieve convergence.

**Table 3:** X-ray diffraction data and structure refinement statistics.

	NAMPT + GNE-618	NAMPT_S165F	NAMPT_S165F + GNE-618	NAMPT_S165F + pRib-GNE-617	NAMPT_S165Y	NAMPT_G217A	NAMPT_ G217V	NAMPT_ G217R	NAMPT_G217R+APO866	NAMPT_H191R	NAMPT_H191R+APO866	NAMPT_S165F + GNE-617
PDB code	4O13	4O14	4O15	4O16	4O17	4O18	4O19	4O1A	4O1B	4O1C	4O1D	4O28
Space group	P2_1_	P2_1_	P2_1_	P2_1_2_1_2	P2_1_	P2_1_	P2_1_	P2_1_	P2_1_	P2_1_	P2_1_	P2_1_
Unit cell	a = 60.6 Å, b = 106.2 Å,c = 82.9 Å α = 90° β = 96.4° γ = 90°	a = 60.3 Å, b = 105.6 Å,c = 83.3 Å α = 90° β = 96.9° γ = 90°	a = 60.4 Å,b = 106.4 Å,c = 83.6 Å α = 90°, β = 96.8°,γ = 90°	A = 86.7 Å,b = 107.4 Å,c = 52.7 Å α = β = γ = 90°	a = 60.2 Å, b = 106.4 Å,c = 83.5 Å α = 90° β = 96.6° γ = 90°	a = 60.3 Å, b = 105.8 Å,c = 82.8 Å α = 90° β = 96.1° γ = 90°	a = 60.6 Å,b = 106.3 Å,c = 83.3 Å α = 90° β = 96.4° γ = 90°	a = 60.7 Å, b = 106.0 Å,c = 82.5 Å α = 90° β = 96.3° γ = 90°	a = 60.6 Å, b = 106.3 Å,c = 83.3 Å α = 90° β = 96.3° γ = 90°	a = 60.4 Å, b = 106.6 Å,c = 82.9 Å α = 90° β = 96.1° γ = 90°	a = 60.7 Å, b = 106.6 Å,c = 83.4 Å α = 90° β = 96.4° γ = 90°	a = 60.3 Å, b = 106.2 Å,c = 83.7 Å α = 90° β = 96.7° γ = 90°
Resolution	50.0–1.75 Å	50.0–1.90 Å	50.0–1.80 Å	50.0–1.79 Å	50.0–1.82 Å	50.0–1.92 Å	50.0–1.75 Å	50.0–1.87 Å	50.0–1.65 Å	50.0–2.10 Å	50.0–1.71 Å	50.0 - 1.98 Å
Total number of reflections	104905 (10471)^1^	77292 (5995)^1^	96336 (9511)^1^	46649 (4589) 1	93025 (9243)^1^	77889 (814)^1^	105718 (10541)^1^	82942 (8311) 1	125279 (12474)^1^	60939 (6125)^1^	113996 (11364)^1^	73761 (7357)^1^
Completeness (%)	99.9 (100)	92.6 (94.2)	99.3 (98.6)	100 (100)	100 (100)	98.9 (100)	100 (100)	99.8 (99.8)	99.9 (99.8)	99.0 (100)	99.9 (100)	100 (100)
Redundancy	3.7 (3.7)	3.6 (3.5)	3.8 (3.7)	6.0 (6.0)	3.8 (3.7)	3.4 (3.3)	3.7 (3.7)	3.6 (3.5)	3.5 (3.1)	3.5 (3.7)	3.7 (3.7)	3.7 (3.7)
I/σ	13.2 (2.0)	18.6 (2.8)	13.5 (2.8)	20.1 (2.1)	11.9 (2.1)	16.0 (2.7)	15.0 (2.8)	9.8 (2.8)	15.4 (2.3)	9.5 (2.4)	14.4 (2.6)	12.5 (2.6)
Rsym^2^	0.108 (0.772)	0.112 (0.497)	0.118 (0.654)	0.080 (0.874)	0.123 (0.821)	0.140 (0.523)	0.085 (0.531)	0.130 (0.583)	0.072 (0.507)	0.116 (0.605)	0.082 (0.525)	0.102 (0.511)
Resolution range	50–1.75 Å	50–1.90 Å	50.0–1.80 Å	5.00–1.79 Å	50–1.82 Å	50–1.92 Å	50.0–1.75 Å	5.00–1.87 Å	50–1.65 Å	50.0–2.10 Å	50.0–1.71 Å	50.0 - 1.98 Å
Rcryst^3^/Rfree^4^	0.195/0.235	0.161/0.201	0.156/0.195	0.178/0.219	0.211/0.258	0.184/0.231	0.153/0.183	0.160/0.203	0.186/0.223	0.239/0.292	0.171/0.208	0.150/0.194
Non-hydrogen atoms	8577	8443	8628	4120	8258	8291	8626	8432	8832	8110	8682	8399
Water molecules	929	858	1050	250	701	737	1024	833	1132	566	1010	794
Average B, Overall	14.12	11.92	11.27	20.38	16.6	13.09	12.88	16.25	11.63	13.08	14.80	13.58
r.m.s.d. bond lengths	0.011 Å	0.005 Å	0.007 Å	0.010 Å	0.012 Å	0.007 Å	0.011 Å	0.007 Å	0.009 Å	0.004 Å	0.011 Å	0.007 Å
r.m.s.d. angles	1.271°	0.907°	1.041°	1.200°	1.314°	1.030°	1.245°	1.030°	1.182°	0.823°	1.270°	1.056°
Ramachandran (C/A/G/D)	0.907/0.093/0/0	0.915/0.085/0/0	0.914/0.086/0/0	0.915/0.085/0/0	0.907/0.092/0/0.001	0.902/0.098/0/0	0.900/0.100/0/0	0.913/0.087/0/0	0.903/0.096/0.001/0	0.901/0.099/0/0	0.920/0.079/0.001/0	0.915/0.085/0/0

1 Values in parentheses for are of the highest resolution shell

2 Rsym  =  Σ|I_hi_ - I_h_|/ΣI_hi_, where I_hi_ is the scaled intensity of the *i*th symmetry-related observation of reflection *h* and I_h_ is the mean value.

3 Rcryst  =  Σ_h_|F_oh_ - F_ch_|/Σ_h_F_oh_, where F_oh_ and F_ch_ are the observed and calculated structure factor amplitudes for reflection *h*.

4 Value of Rfree is calculated for 5% randomly chosen reflections not included in the refinement.

## Supporting Information

Figure S1 **Resistant cell lines do not re-express NAPRT1 and exhibit smaller decreases in NAD in response to GNE-618.** a) Western blots from whole cell lysates of cell lines selected to grow at the indicated doses of GNE-618 represented as fold over IC_50_ values shown in Table 1, PC = positive control, NC = negative control, b) Total cellular NAD before and after exposure to GNE-618 at 100 fold the IC_50_ of the parental cell line, error bars represent the standard deviation of three replicates, c) percent decrease in cellular NAD, the dashed line marks 90% decrease.
(TIF)

Figure S2 **GNE-617 in complex with wild-type and S165F NAMPT.** NAMPT protein is depicted in ribbons diagram, with wild-type in brown and S165F in cyan. The ligands, GNE-617, are shown in sticks, with orange from the wild-type structure and blue from the S165F mutant structure.
(TIF)
