# Supplementary figures and images for: Structural Basis for Resistance to Diverse Classes of NAMPT Inhibitors
Source: PLoS One. 2014 Oct 6;9(10):e109366. doi: 10.1371/journal.pone.0109366 (PMC4186856; doi:10.1371/journal.pone.0109366)

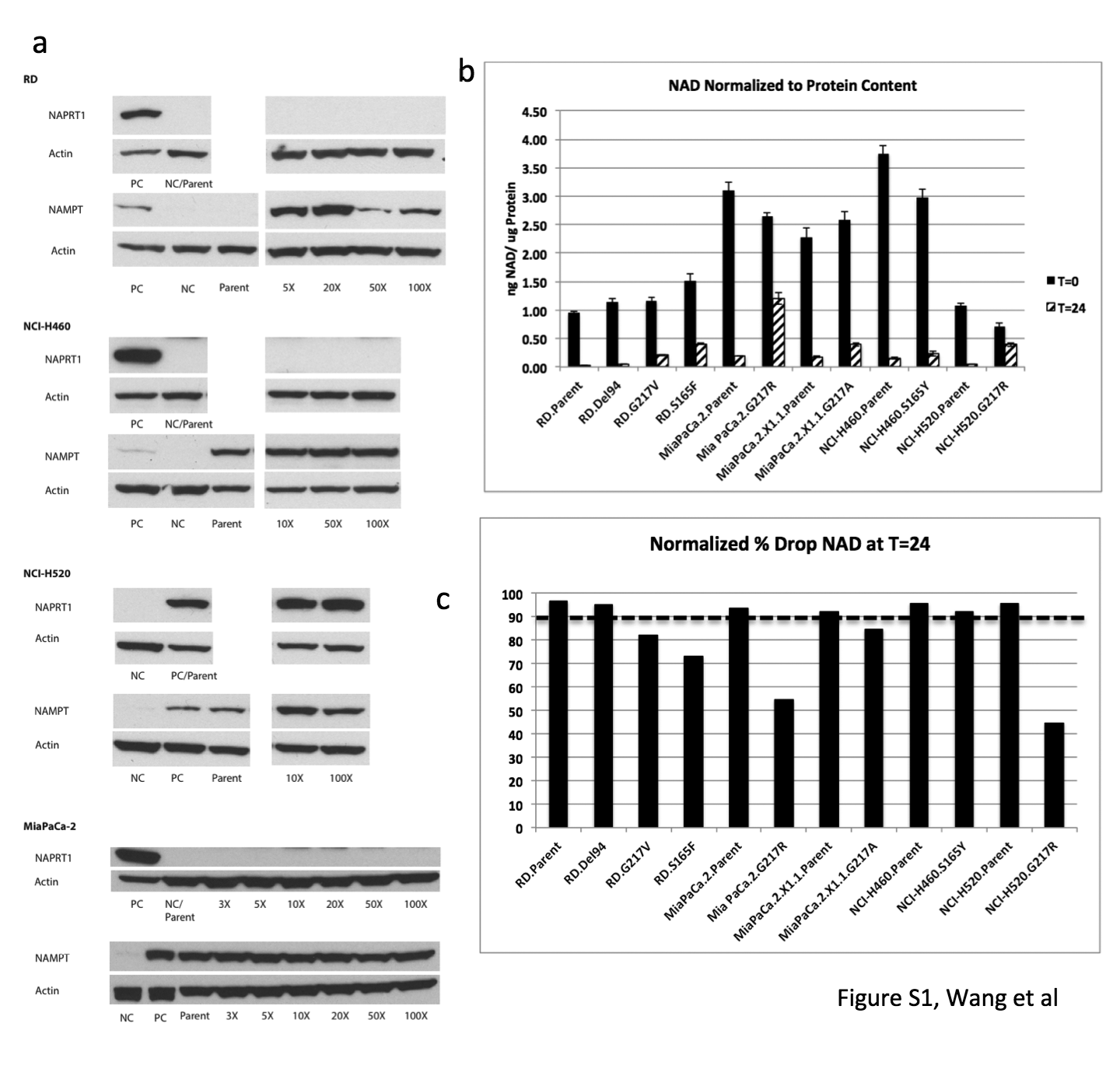

Supplement: Figure S1 — Resistant cell lines do not re-express NAPRT1 and exhibit smaller decreases in NAD in response to GNE-618. a) Western blots from whole cell lysates of cell lines selected to grow at the indicated doses of GNE-618 represented as fold over IC50 values shown in Table 1, PC = positive control, NC = negative control, b) Total cellular NAD before and after exposure to GNE-618 at 100 fold the IC50 of the parental cell line, error bars represent the standard deviation of three replicates, c) percent decrease in cellular NAD, the dashed line marks 90% decrease. (TIF) [file pone.0109366.s001.tif]

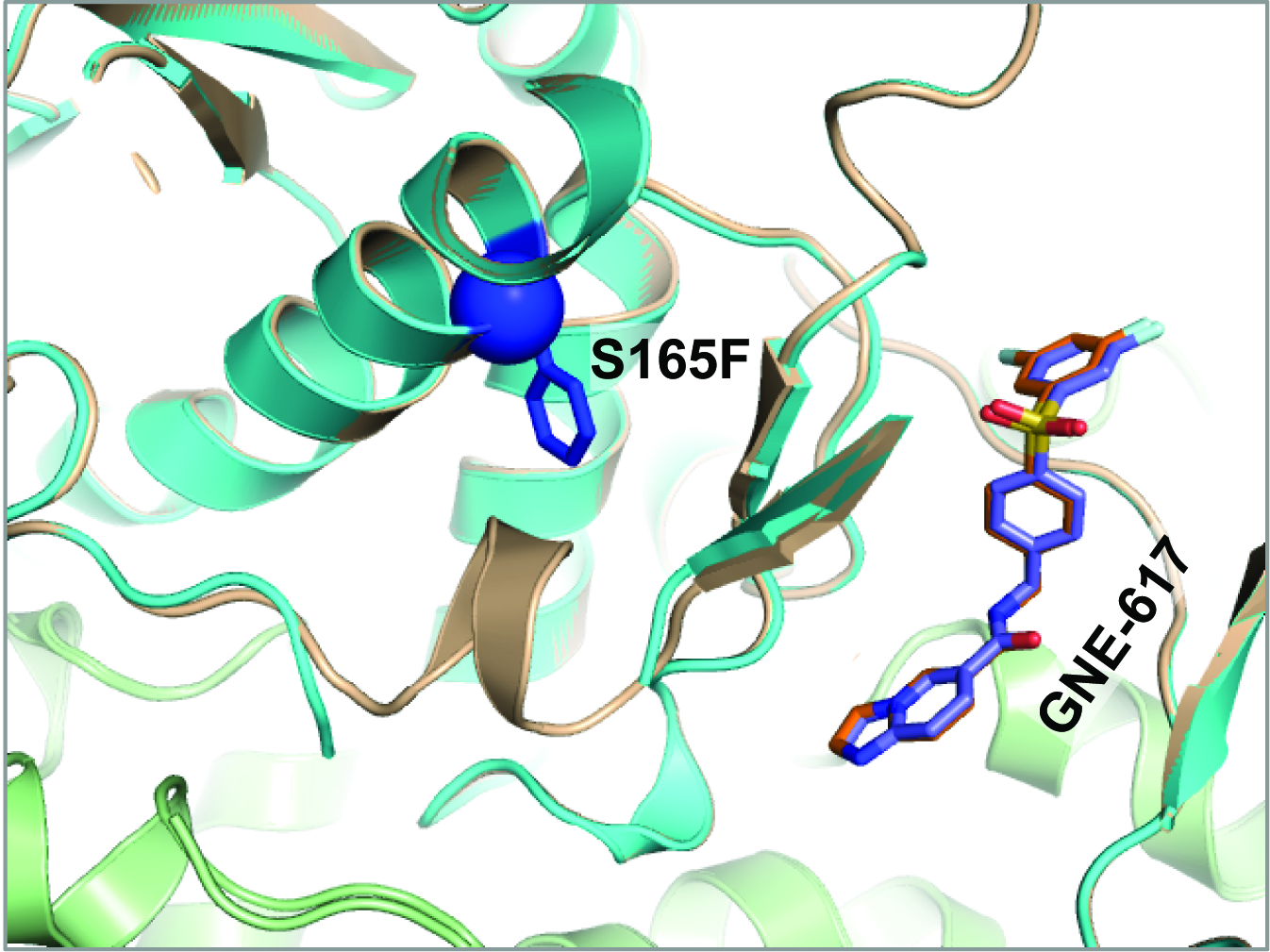

Supplement: Figure S2 — GNE-617 in complex with wild-type and S165F NAMPT. NAMPT protein is depicted in ribbons diagram, with wild-type in brown and S165F in cyan. The ligands, GNE-617, are shown in sticks, with orange from the wild-type structure and blue from the S165F mutant structure. (TIF) [file pone.0109366.s002.tif]
